# Ability of Swept-Source Optical Coherence Tomography to Detect Retinal and Choroidal Changes in Patients with Multiple Sclerosis

**DOI:** 10.1155/2018/7361212

**Published:** 2018-11-13

**Authors:** Elena Garcia-Martin, Laura Jarauta, Elisa Vilades, Jose Ramon Ara, Jesus Martin, Vicente Polo, Jose Manuel Larrosa, Luis Emilio Pablo, Maria Satue

**Affiliations:** ^1^Ophthalmology Department, Miguel Servet University Hospital, Zaragoza, Spain; ^2^IIS-Aragon. Aragon Institute for Health Sciences, Zaragoza, Spain; ^3^Neurology Department, Obispo Polanco Hospital, Teruel, Spain; ^4^Neurology Department, Miguel Servet University Hospital, Zaragoza, Spain

## Abstract

**Purpose:**

To evaluate the ability of new swept-source (SS) optical coherence tomography (OCT) technology to detect changes in retinal and choroidal thickness in patients with multiple sclerosis (MS).

**Methods:**

A total of 101 healthy and 97 MS eyes underwent retinal and choroidal assessment using SS Triton OCT (Topcon). Macular thickness and peripapillary data (retinal, ganglion cell layer (GCL+, GCL++) and retinal nerve fiber layer (RNFL) thickness) were analyzed, including choroidal thickness evaluation.

**Results:**

Significant macular thinning was observed in all ETDRS areas (*p* < 0.001) in MS patients. Peripapillary retinal, RNFL, and GCL ++ thickness showed a significant reduction in patients in all sectors (*p* < 0.001) except in the nasal quadrant/sector (*p* > 0.05). GCL+ measurements were found to be reduced in the nasal (*p*=0.003), inferonasal (*p*=0.045), and temporal (*p*=0.001) sectors and total thickness (*p* < 0.001). Choroidal thickness was reduced in the outer macular ring in MS patients compared with controls (*p*=0.038).

**Conclusion:**

New swept-source technology for OCT devices detects retinal thinning in MS patients, providing increased depth analysis of the choroid in these patients. MS patients present reduced retinal and choroidal thickness in the macular area and reduced peripapillary retinal, RNFL, and GCL thickness.

## 1. Introduction

Optic nerve atrophy and thinning of the peripapillary retinal nerve fiber layer (RNFL) are two typical findings of patients with multiple sclerosis (MS). Axonal damage already occurs in the early stages of the disease, and its relationship with demyelination is still unclear [1, 2]. Axonal damage is considered to be the main cause of disability in MS [3–5] and can be detected and quantified at the level of the retinal nerve fiber layer (RNFL) using ocular imaging technologies, such as optical coherence tomography (OCT) [5–10]. So far, studies using spectral-domain OCT have revealed that the retina in nonoptic neuritis (non-ON) eyes shows thinner peripapillary RNFL (pRNFL) than healthy controls [4, 11–13].

Digital imaging technologies in ophthalmology have greatly improved in the recent years. The most recent milestone in the development of retina and choroid structural visualization strategies is swept-source (SS) OCT, which overcomes the scattering of light on the choroid thanks to longer wavelengths than those used in SD systems (1,050 nm vs 840 nm) [14]. The scan speed in SS-OCT devices is of 100,000 A scans/sec, providing more accurate three-dimensional images of the retina and choroid [14, 15].

In the present study, we evaluated retinal and choroidal thickness in patients with MS without previous ON episodes (that is, without acute loss of RNFL thickness), using new SS-deep range imaging (DRI) OCT technology and compared obtained measurements with a group of healthy subjects. To the best of our knowledge, this is the first study evaluating MS eyes using new SS-DRI OCT technology.

## 2. Methods

All procedures in this study adhered to the tenets of the Declaration of Helsinki; the experimental protocol was approved by the Ethics Committee of the Miguel Servet Hospital (CEICA), and all participants provided written informed consent to participate in the study.

Patients with definite relapsing-remitting (RR) MS were included in an observational cross-sectional study. The sample size was calculated in order to detect significant differences in the RNFL, assuming an *α* error of 5% and a *β* error of 10%. Based on these calculations, the number of subjects needed was at least 80. A total of 97 eyes of 97 patients and 100 eyes of 100 healthy individuals were evaluated.

The diagnosis of MS was based on the McDonald criteria [16] and confirmed by a neurologist. Related medical records (neurological and ophthalmological) were carefully evaluated, and information about Expanded Disability Status Scale (EDSS) scores, disease duration and subtype, modifying disease treatments, and prior episodes ON (based on the standardized definition of ON using clinical criteria [17]) were recorded. Only patients with relapsing-remitting MS were included in our study. Patients with visual acuity <0.1 (6/60, using the Snellen chart) and intraocular pressure >20 mmHg and/or active MS outbreaks (of any neurologic deficit) in the 6 months preceding enrollment in the study were excluded from the study. The reason to exclude active neurological flare ups was that acute axonal losses did not mask neuronal damage secondary to MS progression (i.e., chronic neurodegeneration). Patients with previous ON history were excluded from the study because we aimed at evaluating the ability of Triton OCT to detect subclinical axonal damage in the RNFL of these patients compared to controls. Axial length was assessed in all individuals. Eyes longer than 25.2 mm and refractive errors ≥5 diopters (D) of spherical equivalent or ≥3 D of astigmatism were excluded from the study.

**Figure 1:**
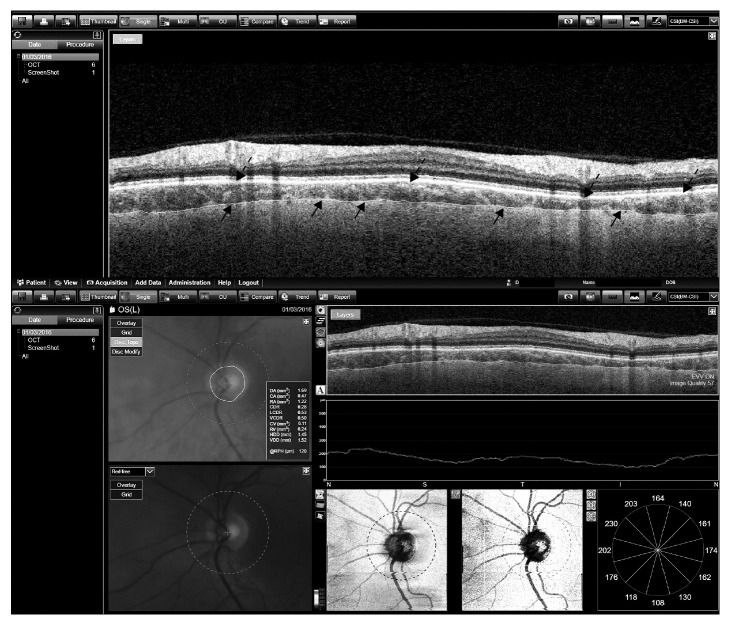
Segmentation of the choroidal plexus of the peripapillary area. Automated segmentation of the peripapillary choroidal plexus and analyzed data provided by Triton OCT in a patient with multiple sclerosis. Arrows indicate the boundaries of the choroidal plexus.

Structural measurements of the retina were obtained using the DRI Triton SS-OCT device (Topcon, Tokyo, Japan) which uses a tunable laser as a light source to provide a 1050 nm centered wavelength. This device reaches a scanning speed of 100,000 A-scans per second, yielding 8 and 20 *μ*m axial and transverse resolution in tissue, respectively. The 3D wide protocol was used for all subjects. This protocol includes a wide scanning range that focuses both in the macular (ETDRS scan) and the peripapillary area (TSNIT). The diameter of the peripapillary area measured with this protocol is 3.40 mm. With the ETDRS scan nine macular areas [18] (which include a central 1 mm circle representing the fovea, and inner and outer rings measuring 3 mm and 6 mm in diameter, respectively), central and average thickness plus macular volume are analyzed; the TSNIT scan provides automated separate measurements of different retinal layers: retinal nerve fiber layer (RNFL) (between the inner limiting membrane (ILM)to the GCL boundaries), ganglion cell layer (GCL) + (between RNFL to the inner nuclear layer boundaries), GCL++ (between ILM to the inner nuclear layer boundaries), and retinal thickness (from the ILM to the retinal pigment epithelium boundaries). The TSNIT provides measurements of the 4 peripapillary quadrants (superior, nasal, inferior, and temporal), 6 sectors (superonasal, superotemporal, nasal, temporal, inferonasal, and inferotemporal), and 12 clock sectors. Additionally, both EDTRS and TSNIT protocols provide automated choroidal thickness measurements (from the Bruch membrane to the choroidal-scleral interface, Figure 1).

In this study, we followed the methods of Satue et al. 2017 [19]. Macular (ETDRS, GCL+, and GCL++) and peripapillary (RNFL, GCL+, and GCL++) thickness were evaluated. Choroidal measurements were obtained for both the macular (ETDRS) and peripapillary area (TSNIT).

All variables were registered in a database created with Excel 2010 (Microsoft Corporation). Modifier variables were age, sex, intraocular pressure, and axial length. Statistical analysis was performed using commercial predictive analytics software (SPSS, version 20.0; SPSS, Inc., Chicago, IL). The normality of the sample distribution was confirmed using the Kolmogorov–Smirnov test (*p* > 0.05). Bonferroni correction for multiple comparisons was applied. Comparisons between patients and controls were calculated using Student's *t* test. *p* value ≤0.05 was considered of statistical significance. Results of calculations are reported as “mean” and “standard deviation.” Only one eye per patient was randomly selected for the study.

## 3. Results

Ninety-seven eyes of 97 RR MS patients with a mean age of 48.46 years (SD = 11.31) and 101 eyes of 101 healthy individuals with a mean age of 47.58 years (SD = 9.37) were included in the study. The male/female ratio was 1/11 in the MS group (8/89) and 1/10 in the control group (9/91). Mean axial length was 23.52 ± 0.6 mm in the control group, and 23.60 ± 0.2 mm in the MS group. Mean age, sex, intraocular pressure, and axial length did not differ significantly between the groups (*p*=0.610, 0.659, 0.865, and  0.522, respectively). Mean disease duration in the group of patients was 7.56 years (SD = 2.66). The median EDSS score was 1.50 (interquartile range (IQR) = 2.30), and all patients suffered from relapsing-remitting MS subtype. No previous acute optic neuritis attack was reported in any of our patients.

Measurements obtained with SS DRI Triton OCT showed reduced retinal thickness in all macular ETDRS sectors in MS patients compared with controls (*p* < 0.001). Macular volume was also significantly reduced in patients (7.92 mm^3^ in controls vs 7.54 mm^3^ in patients, *p* < 0.001). Results can be observed in Table 1.

Significant peripapillary retinal and RNFL thinning was observed in all measured areas (total thickness, quadrants, and sectors, *p* < 0.001) except in the nasal quadrant/sector (Table 2).

The ganglion cell layer as measured from the RNFL to the boundaries of the inner nuclear layer (GCL+) was found to be reduced in the nasal (40.96 *µ*m in controls vs 38.96 *µ*m in patients, *p*=0.003) and temporal (52.31 *µ*m vs 49.20 *µ*m, *p*=0.001) quadrants, in the nasal (40.96 *µ*m vs 38.96 *µ*m, *p*=0.003), inferonasal (37.51 *µ*m vs 35.57 *µ*m, *p*=0.045), and temporal (52.31 *µ*m vs 49.20 *µ*m, *p*=0.001) sectors, and in total thickness (43.20 *µ*m vs 41.21 *µ*m, *p* < 0.001) (Table 2).

Automated measurements of the macular and peripapillary choroidal thickness in MS patients did not show any significant differences compared with healthy controls. However, a clear tendency toward reduced choroidal thickness was observed in the peripapillary area (Table 3). This tendency was not observed in the ETDRS macular choroidal measurements (Supplementary Table 1). A secondary analysis of the ETDRS choroidal thickness was performed due to high standard deviation values. The four areas of the 3 mm inner ETDRS ring (superior, nasal, inferior, and temporal) were assembled and calculated as only one area (inner ring), and the four areas of the 6 mm outer ETDRS ring (superior, nasal, inferior and temporal) were calculated as other unique area (outer ring). The outer ring of the choroidal plexus was found to be significantly reduced in MS patients compared with controls (261.41 *µ*m in controls vs 254.46 *µ*m in patients, *p*=0.038).

## 4. Discussion

In the present study, we evaluated retinal and choroidal changes in MS patients using new SS DRI Triton OCT device. To the best of our knowledge, this is the first study assessing the ability of Swept-source OCT technology to detect retinal changes in MS. Triton SS-OCT analyzed retinal thickness in both the macular and peripapillary area and also two other retinal layers: RNFL and GCL + (both in the peripapillary area). Triton SS-OCT detected significant retinal thinning in all the ETDRS areas in our patients, as well as retinal and RNFL thinning in all sectors of the peripapillary measurements (except in the nasal quadrant/sector). Surprisingly, ganglion cell layer measurements (GCL+) only showed significant reduction in the nasal and temporal quadrants/sectors.

Previous studies using SD-OCT segmentation analysis software demonstrated a reduction of the macular inner retinal layers, including the GCL + IPL, in patients with MS, suggesting ganglion cell loss [20–23]. Recent research demonstrated that GCL measurements as obtained with SD-OCT devices may be a better marker for axonal degeneration in MS compared with RNFL thickness [23]. In a recent study comparing both macular GCL + IPL and peripapillary RNFL thickness, average GCL + IPL was altered more frequently than average peripapillary RNFL, and GCL + IPL thickness demonstrated to have better sensitivity than temporal peripapillary RNFL thickness for detecting retinal thickness changes in patients with MS [12, 24]. Our study did not include macular GCL + thickness. However, in our patients, peripapillary RNFL thickness was altered more frequently than peripapillary GCL measurements (GCL+), and the latter were more reduced in temporal and nasal areas. The fact that the nasal quadrant is affected only in the GCL + in our patients (and not in the RNFL, GCL++) may suggest that, indeed, GCL measurements are somewhat a more subtle marker for neurodegeneration than RNFL thickness. However, more studies with SS-OCT technology including macular GCL + measurements are needed to corroborate these findings.

The choroid (present both in the ocular globe and the central nervous system) is composed of epithelial cells resting on a basal lamina. These epithelial cells produce the cerebrospinal fluid, which has many functions, such as providing mechanical support, a route for some nutrients and removing by-products of metabolism and synaptic activity [25]. The tight junctions located in the choroidal epithelial cells of the central nervous system form the blood-cerebrospinal fluid barrier. Previous atrophy of the choroidal epithelial cells and thickening of the basement membrane were observed in the central nervous system's choroidal plexus of patients with other neurodegenerative diseases, such as Alzheimer's disease [25]. In MS, an inflammatory disease, pathogenic autoreactive T lymphocytes may migrate through the blood-cerebrospinal fluid barrier of the choroid into the central nervous system [25]. Few previous studies focused on ocular choroidal thickness measurements in MS using OCT imaging, and none of them included SS technology. Esen et al. demonstrated, using enhanced depth imaging (EDI) SD OCT, that the subfoveal choroidal plexus in these patients was significantly reduced compared with healthy individuals, and this reduction was associated with disease duration [26]. Our patients showed significant reduced choroidal thickness in the assembled outer ring of the macular area, but not when the 9 separate EDTRS areas were compared. Additionally, the peripapillary choroidal plexus seemed to be diminished in our patients compared with controls (although this finding was not significant). Choroidal thickness is influenced by the axial length of the eye. Both refractive errors and axial length were evaluated in our study and did not differ significantly between patients and controls. Recent research comparing SD-OCT and SS-OCT choroidal measurements in healthy and high myopic subjects demonstrated that SS-OCT measurements provide better quality of choroidal images, which allows higher rates of measurement of this layer [15, 27]. However, choroidal measurements in our study were automatically obtained, which could add segmentation artefacts, but also decrease bias induced by manual measurement. In our opinion, more studies comparing SD-OCT and SS-OCT choroidal measurements in MS patients are needed to corroborate our findings.

A possible limitation is that subclinical glaucomatous eyes might have been included in the study, despite all participants (MS and controls) being evaluated for IOP levels (but not for glaucomatous changes in perimetry). It is possible that both the patient and the control group of subjects contained subclinical glaucomatous eyes. However, since this would have randomly affected both comparative groups, we do not consider this to alter significantly the results of our study. Another possible limitation is that the quality of some of the scans would have caused the choroidal thickness in the MS to be diminished when automated segmentation was applied. Nevertheless, we checked the quality of the scans immediately after acquisition, all quality scores were >55 in both groups, and no statistical difference between the MS and the control group was observed (*p* > 0.05)

## 5. Conclusions

New swept-source technology for OCT devices detects macular thinning and peripapillary retinal, RNFL, and GCL reduction in MS patients, providing increased and more detailed evaluation of the choroid in these patients. Based on our findings, SS-OCT devices could be used as an alternative tool to SD-technology in the routine evaluation of patients with MS. Moreover, since SS-OCT provides increased depth analysis of the choroid in these patients, analyzing the ocular choroidal plexus in MS subjects could add valuable information on the possible affectation of the ocular layers and reflect degeneration in other central nervous system structures. However, similar studies comparing new swept-source and SD OCT technology for the evaluation of MS would still be needed to corroborate our findings.

## Figures and Tables

**Table 1:** Macular retinal thickness and macular volume as measured with swept-source deep range imaging optical coherence tomography Triton in patients with multiple sclerosis and healthy subjects.

Macular thickness	Healthy controls	MS	*p*
Center (*µ*m)	255.38 (35.19)	230.49 (14.54)	**<0.001** ^*∗*^
Inner superior (*µ*m)	320.93 (17.13)	297.08 (19.87)	**<0.001** ^*∗*^
Inner nasal (*µ*m)	323.30 (16.00)	298.93 (13.49)	**<0.001** ^*∗*^
Inner inferior (*µ*m)	318.41 (13.19)	296.88 (13.77)	**<0.001** ^*∗*^
Inner temporal (*µ*m)	306.10 (20.33)	286.54 (12.84)	**<0.001** ^*∗*^
Outer superior (*µ*m)	272.51 (13.87)	263.92 (9.76)	**<0.001** ^*∗*^
Outer nasal (*µ*m)	290.52 (14.84)	274.56 (13.85)	**<0.001** ^*∗*^
Outer inferior (*µ*m)	263.01 (15.75)	252.94 (12.83)	**<0.001** ^*∗*^
Outer temporal (*µ*m)	255.21 (18.31)	248.72 (9.60)	**<0.001** ^*∗*^
Average thickness (*µ*m)	280.41 (13.97)	266.95 (9.83)	**<0.001** ^*∗*^
Volume (mm^3^)	7.92 (0.39)	7.54 (0.27)	**<0.001** ^*∗*^

Numbers indicate mean values with standard deviation between brackets. Bold letters indicate statistical significance. ^*∗*^Significant values after Bonferroni correction for multiple comparisons; MS, multiple sclerosis.

**Table 2:** Peripapillary retinal and ganglion cell layer thickness as measured with swept-source deep range imaging optical coherence tomography Triton in patients with multiple sclerosis and healthy subjects.

	Peripapillary measurements	Healthy controls	MS	*p*
Retinal thickness	Total	291.87 (15.32)	277.75 (16.86)	**<0.001** ^*∗*^
	*Quadrants (x4)*			
	Superior	310.03 (21.53)	292.81 (24.05)	**<0.001** ^*∗*^
	Nasal	262.33 (16.02)	259.02 (14.72)	0.129
	Inferior	313.45 (21.19)	292.03 (21.51)	**<0.001** ^*∗*^
	Temporal	281.53 (12.80)	267.10 (16.70)	**<0.001** ^*∗*^
	*Sectors (x6)*			
	Superonasal	302.64 (27.51)	284.88 (27.06)	**<0.001** ^*∗*^
	Superotemporal	321.31 (20.46)	303.05 (25.56)	**<0.001** ^*∗*^
	Nasal	267.47 (16.33)	262.17 (14.60)	0.016
	Inferonasal	310.43 (26.81)	289.10 (27.09)	**<0.001** ^*∗*^
	Inferotemporal	323.19 (21.25)	300.61 (23.25)	**<0.001** ^*∗*^
	Temporal	281.53 (12.80)	267.10 (16.70)	**<0.001** ^*∗*^

RNFL thickness	Total	104.74 (11.38)	94.60 (14.40)	**<0.001** ^*∗*^
	*Quadrants (x4)*			
	Superior	126.03 (18.03)	112.39 (19.36)	**<0.001** ^*∗*^
	Nasal	76.51 (11.02)	78.97 (14.04)	0.166
	Inferior	139.40 (19.71)	120.23 (20.99)	**<0.001** ^*∗*^
	Temporal	76.93 (12.43)	66.79 (16.64)	**<0.001** ^*∗*^
	*Sectors (x6)*			
	Superonasal	117.01 (25.30)	101.49 (21.42)	**<0.001** ^*∗*^
	Superotemporal	138.00 (18.35)	124.48 (24.93)	**<0.001** ^*∗*^
	Nasal	83.12 (12.00)	83.25 (14.12)	0.966
	Inferonasal	138.63 (27.54)	119.83 (28.49)	**<0.001** ^*∗*^
	Inferotemporal	147.17 (22.60)	126.31 (26.18)	**<0.001** ^*∗*^
	Temporal	76.93 (12.43)	66.79 (16.64)	**<0.001** ^*∗*^

GCL + thickness	Total	43.20 (4.06)	41.21 (3.60)	**<0.001** ^*∗*^
	*Quadrants (x4)*			
	Superior	40.72 (5.39)	39.49 (4.29)	0.076
	Nasal	40.96 (4.36)	38.96 (5.15)	**0.003**
	Inferior	38.81 (5.39)	37.16 (5.73)	0.036
	Temporal	52.31 (7.02)	49.20 (6.03)	**0.001** ^*∗*^
	*Sectors (x6)*			
	Superonasal	41.94 (6.29)	41.37 (4.93)	0.481
	Superotemporal	39.98 (7.54)	38.29 (6.01)	0.083
	Nasal	40.46 (4.32)	38.53 (4.80)	**0.003**
	Inferonasal	37.51 (6.36)	35.57 (7.29)	**0.045**
	Inferotemporal	40.39 (7.03)	38.91 (7.10)	0.139
	Temporal	52.31 (7.02)	49.20 (6.03)	**0.001** ^*∗*^

Numbers indicate mean values with standard deviation between brackets. Bold letters indicate statistical significance. ^*∗*^Significant values after Bonferroni correction for multiple comparisons; MS, multiple sclerosis; RNFL, retinal nerve fiber layer; GCL, ganglion cell layer.

**Table 3:** Macular and peripapillary choroidal thickness as measured with swept-source deep range imaging optical coherence tomography Triton in patients with multiple sclerosis and healthy subjects.

	Healthy controls	MS	*p*
*Macular choroidal thickness*			
Inner ring	278.85 (100.14)	276.41 (86.10)	0.855
Outer ring	261.41 (103.46)	254.46 (79.69)	**0.038**

*Peripapillary choroidal thickness*			
Total	172.44 (94.02)	165.91 (83.22)	0.603
*Quadrants (x4)*			
Superior	193.73 (101.97)	174.55 (79.61)	0.140
Nasal	167.08 (87.91)	164.66 (79.85)	0.838
Inferior	153.01 (101.26)	150.24 (98.71)	0.844
Temporal	175.93 (97.12)	174.20 (93.97)	0.898
*Sectors (x6)*			
*Superonasal*	194.70 (103.29)	171.19 (78.79)	0.072
Superotemporal	196.39 (106.92)	179.33 (82.15)	0.208
Nasal	167.61 (88.95)	164.21 (80.33)	0.777
Inferonasal	150.04 (100.58)	147.76 (100.30)	0.872
Inferotemporal	154.01 (103.59)	151.39 (101.97)	0.857
Temporal	175.93 (97.12)	174.20 (93.97)	0.898

All measurements are in microns. Each of the four macular areas in the 3 mm and 6 mm ring of the ETDRS are assembled into one unique inner and outer ring, due to elevated standard deviation. Bold letters indicate statistical significance. MS, multiple sclerosis.

## Data Availability

Raw data used to support the findings of this study are available from the corresponding author upon request.

## Supplementary Materials

Supplementary Materials Supplementary Table 1: Macular choroidal thickness as measured with Triton optical coherence tomography, in patients with multiple sclerosis and controls.
