# Supplementary material for: Ability of Swept-Source Optical Coherence Tomography to Detect Retinal and Choroidal Changes in Patients with Multiple Sclerosis
Source: J Ophthalmol. 2018 Nov 13;2018:7361212. doi: 10.1155/2018/7361212 (PMC6258108; doi:10.1155/2018/7361212)
Supplement: Supplementary Materials — Supplementary Table 1: Macular choroidal thickness as measured with Triton optical coherence tomography, in patients with multiple sclerosis and controls. [file 7361212.f1.docx]

| **Macular choroidal thickness** | **healthy controls** | **MS** | **p** |
| --- | --- | --- | --- |
| Center | 284.32 (103.92) | 281.47 (92.42) | 0.839 |
| Inner superior | 289.07 (98.06) | 282.92 (89.34) | 0.646 |
| Inner nasal | 272.15 (107.69) | 264.6 (88.37) | 0.591 |
| Inner inferior | 279.28 (107.35) | 279.3 (90.13) | 0.999 |
| Inner temporal | 274.9 (97.41) | 278.83 (90.32) | 0.769 |
| Outer superior | 291.81 (105.92) | 273.72 (84.31) | 0.186 |
| Outer nasal | 223.38 (111.71) | 219.02 (90.41) | 0.763 |
| Outer inferior | 272.62 (116.19) | 263.27 (83.96) | 0.519 |
| Outer temporal | 257.81 (93.70) | 261.82 (81.33) | 0.748 |
| Average | 265.93 (102.24) | 260.09 (80.90) | 0.657 |
